# Testing for the Dual-Route Cascade Reading Model in the Brain: An fMRI Effective Connectivity Account of an Efficient Reading Style

**DOI:** 10.1371/journal.pone.0006675

**Published:** 2009-08-18

**Authors:** Jonathan Levy, Cyril Pernet, Sébastien Treserras, Kader Boulanouar, Florent Aubry, Jean-François Démonet, Pierre Celsis

**Affiliations:** 1 Institut National de la Santé et de la Recherche Médicale (INSERM), Imagerie Cérébrale et Handicaps Neurologiques UMR 825, CHU Purpan, Toulouse, France; 2 Université de Toulouse, UPS, Toulouse, France; 3 SFC Brain Imaging Research Centre, Division of Clinical Neurosciences, Western General Hospital, Edinburgh, Scotland, United Kingdom; 4 Donders Institute for Brain, Cognition and Behaviour, Nijmegen, The Netherlands; 5 Radboud University Nijmegen, Nijmegen, The Netherlands; University of Granada, Spain

## Abstract

Neuropsychological data about the forms of acquired reading impairment provide a strong basis for the theoretical framework of the dual-route cascade (DRC) model which is predictive of reading performance. However, lesions are often extensive and heterogeneous, thus making it difficult to establish precise functional anatomical correlates. Here, we provide a connective neural account in the aim of accommodating the main principles of the DRC framework and to make predictions on reading skill. We located prominent reading areas using fMRI and applied structural equation modeling to pinpoint distinct neural pathways. Functionality of regions together with neural network dissociations between words and pseudowords corroborate the existing neuroanatomical view on the DRC and provide a novel outlook on the sub-regions involved. In a similar vein, congruent (or incongruent) reliance of pathways, that is reliance on the word (or pseudoword) pathway during word reading and on the pseudoword (or word) pathway during pseudoword reading predicted good (or poor) reading performance as assessed by out-of-magnet reading tests. Finally, inter-individual analysis unraveled an efficient reading style mirroring pathway reliance as a function of the fingerprint of the stimulus to be read, suggesting an optimal pattern of cerebral information trafficking which leads to high reading performance.

## Introduction

The theoretical framework of the dual-route cascade (DRC) model enables us to specify the preserved and damaged reading ‘modules’ of neurological patients, and allows us to make quantitative predictions about reading performance in normal readers [1], [2], [3]. The model postulates the existence of two distinct but interactive routes for the processing of written language: the lexical (also called direct) route would process frequent and orthographically irregular words but would fail to do so for unfamiliar words or pseudowords. By contrast, the non-lexical/orthographic (also called indirect) route would process all pseudo- and real words that obey grapheme-to-phoneme (G-P) conversion rules but would fail to produce accurate responses to words that violate these rules, that is, irregular words. Acquired dyslexia, i.e. a selective reading impairment after brain damage in a previously skilled reader, provides direct evidence for these two routes. Patients with acquired surface dyslexia present with left infero-temporal lesions and are very poor at reading irregular words (which are transformed by regularization) whereas their ability to read or spell pseudowords or regular words is unimpaired (e.g. [4]). Semantic dementia patients who suffer from a loss of semantic knowledge also present with lesions to left temporal regions, and are also often poor at reading irregular words ([5], see [6] for a review). By contrast, patients with acquired phonological dyslexia are poor at reading pseudowords whereas their word reading is relatively spared (e.g. [7]), possibly resulting from an impairment in G-P conversion which relies mainly on left inferior-parietal and left infero-frontal regions [8], [9].

This double dissociation between pseudoword reading and irregular word reading suggests that pseudoword reading depends more on the dorsal pathway (parietal cortex) whereas irregular word reading depends more on the ventral pathway (occipito-temporal cortex). In addition, frequent regular words would be processed via both the ventral and dorsal pathways [2]. However, because brain lesions are often extensive and heterogeneous, it is difficult to establish the precise functional anatomical correlates of the lexical and non-lexical routes. Neuroimaging studies have not been more successful in establishing the neural correlates of these two routes [8], [9] as pseudowords and real-words recruit the same neural areas [9], [10], [11], [12]. Alternatively, making inferences on the effective connectivity between reading areas may circumvent this spatial overlap problem and be more suitable for revealing neuro-functional links underlying the DRC model while teasing apart different theoretical viewpoints [13]. In the present study, we focused on the connectivity between posterior reading areas during pseudoword-reading and highly frequent and imageable regular word-reading.

The first aim of this study was to compute effective connectivity between prominent reading areas and to look for plausible connective dissociations during word and pseudoword reading. The second aim of this study was to investigate whether the word/pseudoword dissociation in neural connectivity could account for reading performances.

**Figure 1:**
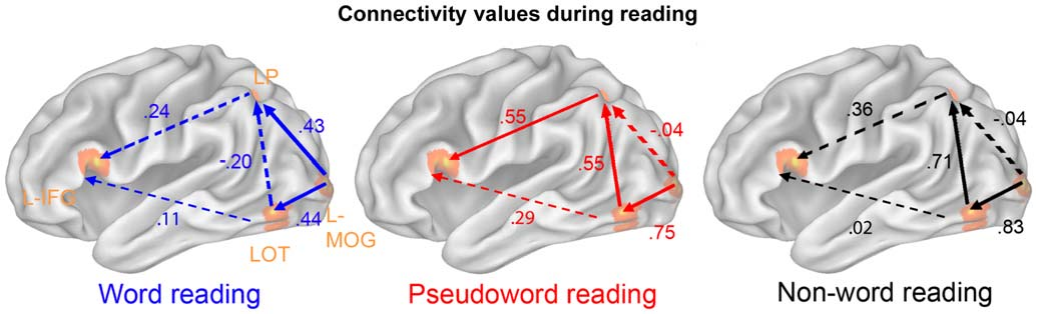
Connectivity values between prominent reading areas. Significant (full arrows) and non-significant (hashed arrows) connectivity values between prominent areas (overlaid on inflated Colin brain atlas anatomical images) during the reading of words (blue), pseudowords (red) and non-words (black). L-MOG, left middle occipital gyrus; LOT, left occipito-temporal junction; LP, left parietal cortex; L-IFG, left inferior frontal gyrus.

To pinpoint the various processing stages (visual, orthographic, phonological) underlying the neural network of reading [14], BOLD signal changes were previously measured using fMRI and connectivity values were computed from 15 subjects who passively viewed eight linguistic (and linguistic-like) stimulus-categories: (a) Single-pseudoletters, (b) 5-pseudoletters, (c) Single-letters, (d) 3-letter strings (consonants), (e) 5-letter strings (consonants), (f) Syllables (3- letters, single-syllable), (g) Words (5-letters, 2-syllables) and (h) Pseudowords (5-letters, 2-syllables) (see example of stimuli on Figure S1). This design allowed us to perform a series of conjunctions over these categories, thereby isolating the following processing stages: visual (all eight stimulus categories), orthographic (c-h) and phonological (f-h). Analyses brought up significant effects bridging brain areas with reading-related processing steps. Results were as follows: the middle occipital gyrus (MOG) activity was consistent with visual processing (left MOG, BA 19 at -32 -91 10), the left occipito-temporal junction (LOT) with letter-string (orthographic) processing (left MOG, BA 37 at -46 -68 -5), the left parietal cortex (LP) with orthographic-phonological transcoding (precuneus, BA 7 at -24 -50 43) and the left inferior frontal gyrus (IFG) with phonological processing (BA 45 at -51 18 14) (see Figure 1). In the current connectivity model, the four ROIs were defined by 3×3×3 mm spheres centered at the above coordinates. These prominent reading areas which were revealed by our own previous work [12] are consistent with many independent lines of research over the years that we reviewed. We previously demonstrated that a connectivity model based on these 4 areas that uses forward connections only, can account for all stimuli to be seen or read [12]. Here, we focused on word and pseudoword reading only and tested for the differential use of the posterior paths. It is of importance to note the posterior localization of the LOT in this study (left MOG, BA 37 at -46 -68 -5) which reflects the initial sub-lexical analysis of written words (thus mainly of sub-word stimuli such as pseudowords) [8], [9], [15], [16], [17], [18], [19], [20] whereas activation of more anterior parts of LOT reflects lexico-semantic processes [12], [16], [17], [20], [21], [22], [23], [24]. With respect to these considerations (for a more encompassed and detailed review c.f. the Discussion section) and the aforementioned double dissociation between surface and phonological acquired dyslexia, we hypothesized that (regular) word reading would rely equally on the MOG→LOT (ventral) and MOG→LP (dorsal) paths (for both sub-lexical and G-P conversion) whereas pseudoword reading would rely on the dorsal path, only after sub-lexical processing in the LOT (MOG→LOT→LP).

To investigate if connectivity values predict participants' reading skills, path coefficients during word and pseudoword reading were used to predict out-of-the scanner performances in (i) lexical (regular words) and non-lexical (pseudowords) reading, and in (ii) text reading. Furthermore, leaning on previous behavioral observations that showed that skilled readers shift between reading styles (non-lexical vs. lexical pathways) in an automated manner depending on the stimuli ‘fingerprint’ (lexicality, transparency, frequency, imageability; [25]), we hypothesized that the difference in connectivity values for the labeled ‘pseudoword pathway’ and ‘word pathway’ would predict the corresponding performances. The fact that explicit tasks during reading induce changes in the use of language neural pathways [26], [27], [28] also supports the hypothesis that reading styles may be reflected by the preferential reliance on a neural pathway.

Here, connectivity results provide strong support to the little neuropsychological evidence suggesting an anatomical segregation of the lexical and non-lexical reading routes. However, at variance with previous brain models of reading pathways, our data suggest a more complex scheme for routing information such that the phonology-related ‘dorsal’ link between the parietal and the frontal nodes of the network can be directly accessed from the extrastriate lateral visual cortex by frequent regular words while a posterior ventral ‘detour’ pathway is accessed first during pseudoword reading. In addition, the results show that some subjects who relied inappropriately on the word pathway during pseudoword reading (i.e. incongruent reliance), and vice versa, show poor reading performance, whereas (congruent) reliance on the word pathway during word reading and likewise for pseudowords is predictive of high reading ability.

## Results

### Dissociating word and pseudoword reading

Estimated connectivity values provided an excellent account of the measured data for word (p>0.99; RMSEA<10^−3^) and pseudoword (p>0.67; RMSEA<10^−3^) reading, but also during the processing of the other categories (p-values in Table S1). Note that pseudoword reading data from one subject (number 15) did not fit the connectivity model (p = 0.04; Table S2) and were therefore discarded. To investigate the influence of stimulus-categories on the paths within the network, a stacked model approach was used. This approach consists in comparing a ‘free’ model in which all paths are allowed to vary between two conditions, to a ‘restricted’ model in which the tested path is forced to be equal across conditions.

For the first focus of the study, we applied a first analysis (between paths) in which, differences between path's coefficients were tested for each category. This allowed us to explore whether, for a given stimulus category (words or pseudowords) one path of the network was more involved than the other. For word reading, no difference could be observed between MOG → LOT and MOG → LP (p = .92). In addition, the MOG→LP, MOG→LOT and LP→IFG paths were all significantly more involved than the LOT→LP path (see Table 1), and a close-to-significant effect (p = .07) was observed between LOT → IFG and LOT → LP. These two sets of results thus suggest that during word reading, information traffics in parallel in the ventral and dorsal pathways. For pseudowords, significant differences were observed between MOG → LOT and MOG → LP, as well as between LOT → LP and MOG → LP (Table 2). By contrast to word reading, this suggests that information traffics first from MOG to LOT and then is transferred to the dorsal pathway (although LOT → IFG and LP → IFG do not differ).

**Table 1:** Significance (p, χ^2^) between the involvement (β) of paths during word reading.

vs.	MOG→LOT (β = 0.44)	MOG→LP (β = 0.43)	LOT→LP (β = −0.20)	LOT→IFG (β = 0.11)	LP→IFG (β = 0.24)
**MOG→LOT**	-	p = 0.92 (χ^2^ = 0.01)	p = 0.0002 (χ^2^ = 14.14)	p = 0.047(χ^2^ = 3.93)	p = 0.23 (χ^2^ = 1.44)
**MOG→LP**		-	p = 0.003 (χ^2^ = 8.54)	p = 0.068 (χ^2^ = 3.33)	p = 0.27 (χ^2^ = 1.19)
**LOT→LP**			-	p = 0.07 (χ^2^ = 3.19)	p = 0.01 (χ^2^ = 6.35)
**LOT→IFG**				-	p = 0.45 (χ^2^ = 0.57)

**MOG**, left middle occipital gyrus; **LOT**, left occipito-temporal junction; **LP**, left parietal cortex; **IFG**, left inferior frontal gyrus.

**Table 2:** Significance (p, χ^2^) between the involvement (β) of paths during pseudoword reading.

vs.	MOG→LOT (β = 0.75)	MOG→LP (β = −0.04)	LOT→LP (β = 0.55)	LOT→IFG (β = 0.29)	LP→IFG (β = 0.55)
**MOG→LOT**	-	p = 0.0001(χ^2^ = 18.95)	p = 0.27(χ^2^ = 1.2)	p = 0.0004(χ^2^ = 12.76)	p = 0.11 (χ^2^ = 2.6)
**MOG→LP**		-	p = 0.05 (χ^2^ = 3.8)	p = 0.077 (χ^2^ = 3.13)	p = 0.002 (χ^2^ = 9.53)
**LOT→LP**			-	p = 0.14 (χ^2^ = 2.14)	p = 1 (χ^2^ = 0)
**LOT→IFG**				-	p = 0.14 (χ^2^ = 2.14)

**MOG**, left middle occipital gyrus; **LOT**, left occipito-temporal junction; **LP**, left parietal cortex; **IFG**, left inferior frontal gyrus.

Furthermore, the DRC model postulates distinct routes for lexical and non-lexical reading. Hence, to pinpoint selective pathways as a function of stimulus category, we applied a second analysis (between conditions) to test for differences in connection strength between word and pseudoword reading. This analysis did not aim to control for exclusivity of paths as above, but rather to compare paths' contribution to the model between conditions. This analysis revealed a significantly stronger implication of MOG→LP during word reading than during pseudoword reading (β = 0.43 vs. β = −0.04, χ^2^ = 5.3, p = 0.02) (Figure 1). By contrast, connectivity coefficients of MOG→LOT, LOT→LP and LP→IFG were significantly more robust during pseudoword reading than during word reading (MOG→LOT β = 0.75 vs. β = 0.44, χ^2^ = 5.15, p = 0.02; LOT→LP β = 0.55 vs. β = −0.20, χ^2^ = 5.15, p = 0.0003; note however the χ^2^ = 3.83 and p = 0.05 for LP→IFG β = 0.55 vs. β = 0.24). No significant effect was observed for LOT→IFG (β = 0.29 vs. β = 0.11, χ^2^ = 1.33, p = 0.25). These results thus consolidate our interpretation of a ‘detoured’ route for pseudowords and also highlight the special role of MOG → LP in word vs. pseudoword processing.

In summary, the path MOG→LOT is preferentially used during pseudoword reading while the path LOT→LP is exclusively used during pseudoword reading. Thus, and for ease of explication MOG→LOT→LP is referred to hereafter as the ‘pseudoword pathway’. By contrast, words revealed a preferential (and exclusive) direct connection to LP (MOG→LP), hereafter referred to as the ‘word pathway’, although the ventral (MOG → LOT → IFG) is also likely to be involved. We now focus on the posterior triangle (MOG – LOT – LP) since no difference between LOT → IFG and LP → IFG was observed for words and pseudowords.

### Role of the posterior ‘reading triangle’

Focusing on the posterior triangle MOG – LOT – LP which accounted for the strongest dissociation effects between word and pseudoword reading, we additionally tested for differences between paths during non-word reading (i.e. 5-consonant string) as well as for differences between categories for each path. This additional analysis allowed us to determine whether this ‘mediator triangle’ mirrored visual-orthographic or orthographic-phonological processing.

Overall, non-word (5-consonants without orthographic regularities, phonological units, and lexical entries) reading elicited the same pattern of connectivity as pseudowords did (i.e. 5-letters with orthographic regularities and phonological units but no lexical entries) and the same differences were observed in comparison to words (5-letters with orthographic regularities phonological units and lexical entries - Figure 1, right panel). In details, we observed that: (i) MOG→LOT and LOT→LP were equally involved in non-word processing (β = 0.83 vs. β = 0.71, χ^2^ = 0.50, p = 0.52), (ii) and both significantly more involved than MOG→LP (MOG→LOT β = 0.83 vs. MOG→LP β = −0.04, χ^2^ = 26.55, p = 10^−7^; LOT→LP β = 0.71 vs. MOG→LP β = −0.04, χ^2^ = 6.41, p = 0.01); (iii) MOG→LP was significantly more engaged during word reading than during non-word reading (β = 0.43 vs. β = −0.04, χ^2^ = 5.64, p = 0.02); (iv) as a corollary, connectivity coefficients in both MOG→LOT and LOT→LP were significantly more robust during non-word reading than during word reading (MOG→LOT β = 0.83 vs. β = 0.44, χ^2^ = 8.85, p = 0.003; LOT→LP β = 0.71 vs. β = −0.20, χ^2^ = 20.33, p = 10^−6^). However, in contrast to pseudoword reading (words β = 0.24 vs. pseudowords β = 0.55, χ^2^ = 3.83, p = 0.05), connectivity in the LP→IFG path was modulated to the same extent by words and non-words (words β = 0.24 vs. non-words β = 0.36, χ^2^ = 0.40, p = 0.53). To summarize, the fact that non-word processing replicated the effects of pseudowords (vs. words) for the posterior connections only, suggests that the MOG→LOT→LP triangle is implicated in visual-orthographic analysis (but not necessarily in the processing of orthographic regularities) whereas the LP→IFG branch is more involved in orthographic-phonological transcoding, in agreement with previous reports [12], [15], [29].

In a nutshell, as hypothesized, results show that visual information accesses from MOG both the ventral path (to LOT) and the dorsal path (to LP), and that pseudoword reading engages strongly the anterior dorsal path (LP→IFG). However, we demonstrate that the latter involves a detour via the posterior ventral path (LOT) that is likely crucial to orthographic processing. In addition, although words engage both ventral and dorsal pathways, our data suggest a preference for the dorsal one (MOG→LP).

### Predicting reading performances

For the second purpose of our study, we tested whether the above-described double dissociation in neural pathways could predict reading performance as measured outside the scanner. Spearman rank correlation coefficients (p<0.05 - Table 3) were computed between on the one hand, z-scored (Table 4) Word Reading Test (WRT) and Pseudoword Reading Test (PWRT) accuracy scores [30], and “*Alouette-R*” CTL indices [31], and on the other hand, BOLD-derived path coefficients for all modeled connections during word and pseudoword reading (Table 3). As elaborated in the Methods section, the first two tests are markers of impairment in word or pseudoword reading [32], [33], whereas “*Alouette-R*” test is particularly conceived to assess proficiency at rapidly alternating between lexical and non-lexical processing during text reading [31]. For this second part of the study, we thus formulated two hypotheses: (i) scores in WRT and PWRT would negatively correlate with incongruent reliance of pathways, i.e. reliance on the word pathway during pseudoword reading and vice versa, and (ii) CTL (“*Alouette-R*”) scores would positively correlate with congruent reliance of pathways, i.e. reliance on the word pathway during word reading and on the pseudoword pathway during pseudoword reading.

**Table 3:** Spearman correlation coefficients between off-line reading tests and β-values of effective connectivity.

	PW reading	PW reading	PW reading	W reading	W reading	W reading
	*W RT*	*PW RT*	*CTL*	*W RT*	*PW RT*	*CTL*
**MOG → LOT**	0.28	0.18	***0.58***	***−0.70***	***−0.50***	−0.42
**MOG → LP**	−0.22	***−0.52***	−0.04	0.27	−0.03	***0.46***
**LOT → LP**	0	0.10	0.24	***−0.56***	***−0.60***	−0.423
**LOT → IFG**	0.38	0.17	0.40	0.43	0.13	−0.2
**LP → IFG**	0.26	0.05	0.31	0.03	−0.03	0.21

Significant correlations are highlighted in ***italic bold***. **PW reading**, correlation with connectivity values during on-line pseudoword reading; **W reading**, correlation with connectivity values during on-line word reading;**MOG**, left middle occipital gyrus; **LOT**, left occipito-temporal junction; **LP**, left parietal cortex; **IFG**, left inferior frontal gyrus; *WRT*, correlation with the off-line Word reading Response Time test; *PWRT*, correlation with the off-line Pseudoword reading Response Time test; *CTL*, correlation with the off-line speed and precision index used in the “*Alouette-R*” test.

**Table 4:** Individual delta PW, delta W values, as well as scores in the out-of-magnet reading tests.

	W RT	PW RT	CTL	Delta PW	Delta W
1	−0.17	0.20	683.48	−0.16	−0.27
2	−0.49	−0.05	372.96	−0.35	−0.23
3	0.93	0.98	594.68	−0.34	−0.25
4	2.62	1.61	622.9	−0.33	0.12
5	1.72	0.98	653.43	−0.51	0.18
6	2.44	1.92	625.26	−0.47	−0.04
7	0.60	1.36	510	0.18	−0.20
8	0.95	−0.5	491.61	0.18	0.02
9	0.91	0.85	572.93	−0.27	−0.30
10	−0.27	−0.09	436.67	0.34	−0.43
11	2.60	1.95	594	−0.41	−0.08
12	0.92	1.21	589.5	−0.45	−0.07
13	0.59	0.88	582.22	−0.16	−0.07
14	2.18	1.86	570.36	0.09	−0.30
15	1.60	1.32	685.59	0.14	0.14
*mean*	1.14	0.97	572.37	−0.17	−0.12
*sd*	1.03	0.77	87.44	0.28	0.18

**WRT**, Word reading Response Time test (off-line); **PWRT**, Pseudoword reading Response Time test (off-line); **CTL**, speed and precision index used in the “*Alouette-R*” test (off-line); Delta PW, the difference (delta) between connectivity values of MOG→LP and MOG→LOT during on-line pseudoword reading; Delta W, the same during on-line word reading.

**Figure 2:**
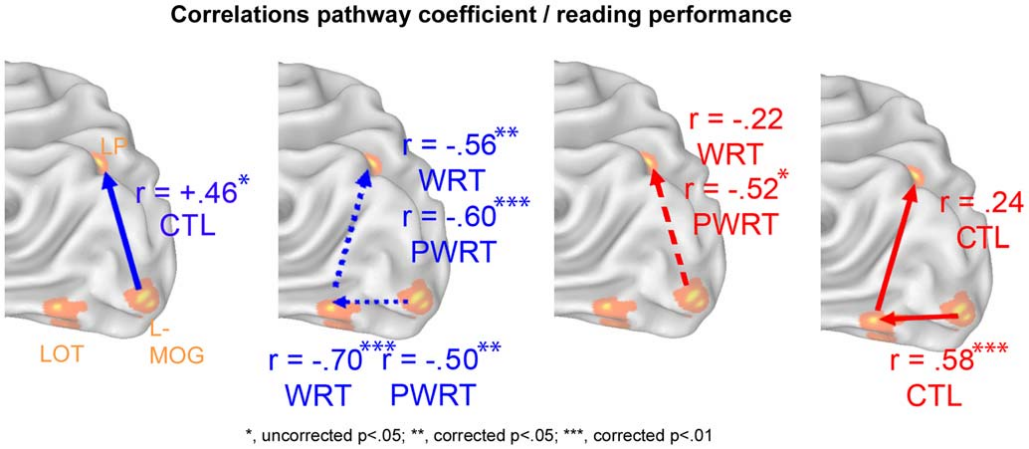
Correlations pathway coefficient/reading performance. Significant (FDR corrected or uncorrected) Spearman positive (full arrows) or negative (hashed arrows) correlation coefficients between off-line reading tests (WRT, PWRT, CTL) and effective connectivity values of on-line word (blue) and pseudoword (red) reading. WRT, Word reading Response Time test; PWRT, Pseudoword reading Response Time test; CTL, speed and precision index used in the “*Alouette-R*” test. L-MOG, left middle occipital gyrus; LOT, left occipito-temporal junction; LP, left parietal cortex; L-IFG, left inferior frontal gyrus.

Testing for the correlations between path coefficients and reading performances, we observed that connectivity coefficients associated with pseudoword reading appraised during (on-line) word reading, negatively correlated with the performance on the *WRT* (**r = −0.70**, corrected for false discovery rate p = 0.002 for MOG→LOT and **r = −0.56**, corrected p = 0.015 for LOT→LP) and on the *PWRT* (**r = −0.50**, corrected p = 0.029 for MOG→LOT and **r = −0.60**, corrected p = 0.009 for LOT→LP) collected out of the scanner (Figure 2, blue hashed arrows). This shows that participants relying on the (posterior) pseudoword pathway (MOG→LOT, LOT→LP) during word reading had poorer performance in word and pseudoword reading. Conversely, connectivity coefficients of the word pathway (MOG→LP) measured during pseudoword reading, negatively correlated with the performance on the *PWRT* (**r = −0.52**, uncorrected p = 0.03) but not the *WRT* (r = −0.22, p = 0.22 - Figure 2, red hashed arrows). Overall, the findings allege that reliance on a neural pathway incongruent with the stimulus to be read predicts poorer reading performance as reflected by these tests.

As for reading skill per se (reflected by CTL index), reliance on the word pathway (MOG→LP) measured during word reading positively correlated (**r = 0.46**, uncorrected p = 0.04) with reading skill (CTL index) (Figure 2, full blue arrows). In a similar vein, reliance on the pseudoword pathway (MOG→LOT) measured during pseudoword reading positively correlated (**r = 0.58**, corrected p = 0.01) with reading skill (Figure 2, full red arrows). This set of findings confirmed the second hypothesis and cogently argues that reliance on a neural pathway congruent with the stimulus to be read predicts reading skill.

**Figure 3:**
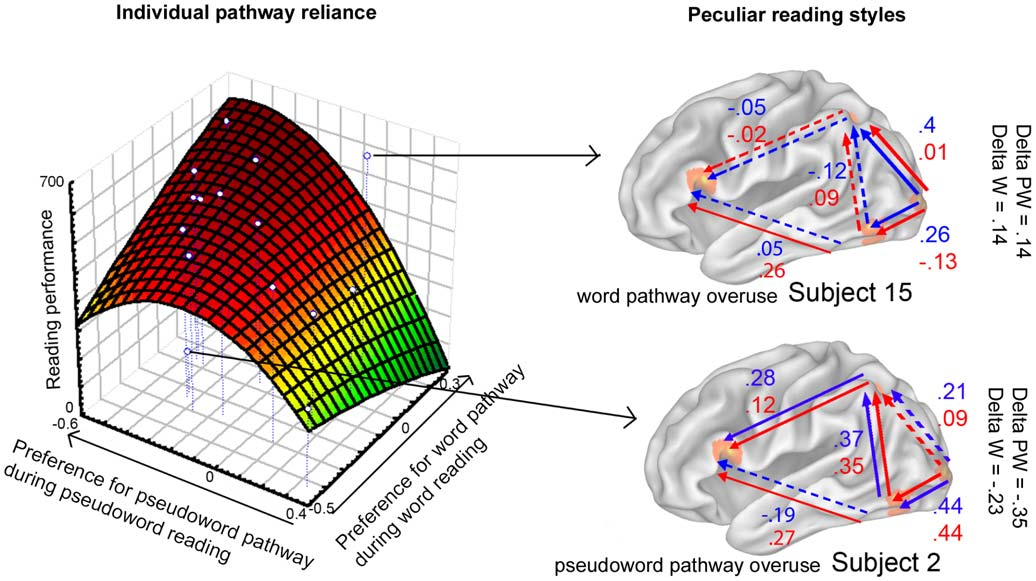
Group and individual pathway reliance. Left Panel: Group trade-off between preferences (delta values) for the word pathway vs. the pseudoword pathway resulting in efficient (dark-red) or deficient (yellow-green) reading performance outside the scanner (CTL index). Right Panel: Subjects with nonconformist reading styles during word (blue) and pseudoword (red) reading which predict their highly-efficient (subject 15) and inefficient (subject 2) reading performance.

Finally, we tested if the preferential use of one or the other pathway, i.e. MOG→LP vs. MOG→LOT, during on-line word vs. pseudoword reading predicted off-line text reading skill (how good one can alternate between reading frequent and infrequent (like pseudowords) words). To this aim, the difference (Delta) between connectivity values of MOG→LP and MOG→LOT during word and pseudoword reading respectively, were computed and correlated with reading skill (CTL indices). We reasoned that subjects with highly positive Delta values (i.e. subjects relying more on the word pathway) during word reading and highly negative Delta values (i.e. subjects relying more on the pseudoword pathway) during pseudoword reading would have the highest scores in reading performance, as evaluated by CTL indices. At first, Spearman correlations only showed a correlation of r = −.57 (p = 0.016) between CTL indices and delta Pseudoword and r = .39 (p = 0.08) between CTL indices and delta Word. Similarly, the multiple regression was not significant (R**^2^** = .28 F(2,11) = 2.11 p = 0.17). However, closer inspection showed that 2 subjects were outliers (see Methods and Supplementary Figure S2). Fitting the data again on the remaining 12 subjects revealed a rank correlation of **r = 0.60** (p = 0.04) for Delta Word/CTL and of **r = − 0.92**, p = 10**^−3^** for Delta Pseudoword/CTL. The regression analysis showed that a linear adjustment to the data explains 85% of the CTL variance (**R = 0.92**, F(2,9) = 25.8, p<0.0002) with a significant contribution of the difference between paths for pseudoword reading (partial correlation t(9) = −5.36, p = 0.0004, R^2^ = −0.87) but not word reading (partial correlation t(9) = 1, p = 0.3, R^2^ = 0.30). A quadratic adjustment of the data explained up to 93% of the CTL variance (**R = 0.967**, F(5,6) = 17.63 p<0.001) suggesting separate optima for each path but an optimal combination of their preferential recruitment depending on the lexicality of stimuli to be processed (Figure 3, left panel). To test if factors, other than connectivity measures in the two putative pathways could explain CTL variance, an augmented model was computed using demographic (age and gender) and educational variables (confidential data not shown here). The new model could explain 90% (F(5,6) = 11.6 p = .004) of the CTL variance but this increase of 5% was not significant (full vs. reduced model, F(4,9) = 1.63 p = .24). Overall, the results confirm our hypothesis that congruent pathway reliance as defined here, namely relying on the word pathway during word reading and on the pseudoword pathway during pseudoword reading, predicts high reading skill.

## Discussion

### Dual route cascade model in the brain

Comparing path coefficients between conditions (reading words vs. pseudowords) and between paths confirmed previous neuropsychological observations of reading impairment as proposed by the DRC model [2]. Between path analyses lead to the following sketchy description of the main stages of information processing during reading (Figure 1): during regular word reading, visual information (MOG) is transmitted both to the ventral path (LOT) and to the dorsal path (LP) with a preference to the latter, information is then transferred to the left IFG. As for pseudoword reading, information is indeed processed via the dorsal path (LP→IFG), but this is done via the posterior ventral path first (MOG→LOT→LP). Additional testing with non-words suggests that the posterior reading triangle is involved in the visuo-orthographic component during reading while LP → IFG is involved in orthographic-phonologic decoding. In a similar vein, between conditions analyses showed that the pathways (originating at the visual cortex) to the posterior ventral and dorsal streams were selective for pseudoword (MOG→LOT→LP) and word (MOG→LP) reading respectively, thus strengthening neuropsychological evidence of the neuroanatomically distinct nature of the routes for pseudoword (non-lexical) and word (lexical) reading. Further consolidating this double dissociation, reliance on these pathways which was incongruent with the stimulus to be read predicts poorer reading performance, whereas congruent reliance on these pathways predicts higher reading skill (Figure 2). The main effects of our results therefore referred to this posterior reading triangle. Analyzing both the anterior dorsal (LP→IFG) and the anterior ventral (LOT→IFG) pathways of the model was less conclusive and therefore less discussed here. The findings allege that for pseudoword reading, LP→IFG is possibly more implicated than LOT→IFG, which is in accordance with its implication in G-P transcoding and phonological processing [12], [15], [29]. It may be that words rely more on the anterior ventral path, but it is also possible that this path originates from a more anterior part of LOT (see further). For the rest of the discussion, we will focus on the posterior reading triangle and how our model fits with the literature.

Our *a priori* model does not rule out the contribution of other (in between) regions to reading. Such spatial-temporal feed-forward cascade is supported by the extensive number of observations in neuroimaging, electro- and magneto-encephalography studies over the years (reviewed in [8], [34], [35], [36]). In short, we do contend that reading is a very complex task involving a very broad network with feed-forward and feed-back loops, yet, we reason that studying its main components during simple tasks using forward model should be enough to highlight main stream differences between conditions. Indeed, in accordance with the principle of parsimony, this forward model fitted well all eight categories of stimuli read or perceived, using the smallest number of parameters (paths) offering a good trade-off between bias and variance.

Although the findings support prior evidence [2], lexical reading is thought to involve more the left-hemispheric ventral pathway whereas non-lexical reading – the dorsal pathway (e.g. [37]); this may appear counter-intuitive with respect to the above findings. A closer investigation of the precise location and functionality of the above regions may resolve this apparent contradiction. First, the ventral pathway is indeed involved in orthographic/lexical processing. Nevertheless, its specificity for lexical processing concerns a more anterior part of the pathway, which is assumed to gate lexico-semantic analysis (see [8], [14] for a review). Previously, following irregular words presentation, effective connectivity between the anterior LOT (y = −42) and the left pars triangularis (IFG) was associated with increased activation in the latter, corroborating with the lexico-semantic role of this anterior pathway [16]. Likewise, the Visual Word Form Area (VWFA) is thought to be attuned to orthographic regularities and thus to be more activated for words or pseudowords than for alphabetical consonant strings [38]. This is suggestive of the more lexico-semantic role of the VWFA and is therefore not surprising that this area is comprised in an anterior segment of the LOT (approximately: -43 -54 -12, in the left fusiform gyrus, BA 37).

Here, however, we isolated a more posterior part of the LOT (left MOG, BA 37 at -46 -68 -5) applying an analysis which isolated (alphabetical) letter-related activations. It is thus more likely that the region here is involved in the initial sub-lexical analysis of written words (see [17]: y = −73) and in the processing of letters strings (sub-words) (see [15]: y = −60; [16]: y = −60; [18]: y = −63; [19]: y = −60; [20]: y = −56 to −64). This fits well with the higher activation of LOT usually observed during pseudoword reading [9], [10], [12], which triggers an excess of sub-word processing relative to word reading. Indeed, pseudoword reading automatically triggers a process of recognition of familiar sub-word segments and a ‘search’ for their original real words, and is therefore slower than any other linguistic stimulus in lexical decision tasks [39]. In a similar vein, visual spelling task (match the spelling of two words from the first vowel onwards) which would probably involve similar processes to those during pseudoword reading, much more than during word reading, indeed yields stronger connectivity in MOG→LOT (y = −72) than in MOG→LP [40]. As such, the posterior LOT is sensitive to ‘low-level’ linguistic stimuli (unfamiliar characters or infrequent letter-combinations), while activation becomes hierarchically more anterior for frequent letters, bigrams, quadrigrams, and the most anterior – for words [20]. Overall, the literature is congruent with our findings if one is to look at sub-regions. It therefore contributes to the recently growing body of evidence of a functional dissociation inside the LOT depending on stimulus type (words, pseudowords, letter strings or unfamiliar characters) suggesting a more perceptual role for the posterior LOT and a more lexico-semantic role for the anterior LOT [12], [16], [17], [20], [21], [22], [23], [24].

Together, in terms of DRC, these observations imply that the ventral path implicated in lexical reading and associated with acquired surface dyslexia actually involves a more anterior segment than the one discussed here. Thus, reading regular words and pseudowords involves information trafficking to the posterior ventral path for letter/sub-word processing. However, this is much more substantial for pseudowords because of the necessity to process its sub-word components. Assuming a strict mapping between brain activation and functional involvement, pseudoword reading mainly relies on the assembling and processing of sub-word units. Information is then likely to be processed in the LP for subsequent G-P mapping (pseudowords), and in the anterior LOT for lexico-semantic access (words).

As for the dorsal path, the LP is often reported in the same studies that report LOT activation for letters [22], [41], [42], [43], [44], [45], thus implying a role in letter processing for both regions. However, in recent years more and more experimental evidence is gained over the more general role of the LOT [12] reflecting an operation common to the processing of words [46], word sub-units [19], pictures [47], objects [48], or stored visual forms and structures in general [49]. Noteworthy, the coordinates of LP in the present study fall mainly in the left superior parietal lobule which together with the left inferior parietal lobule yields greater letter-selectivity than does the LOT [50], [51]. Moreover, this letter-selective cluster in the LP also revealed (i) phonological selectivity (phonological null conjunction), and (ii) a positive activity gradient (linear contrast) as a function of the number of orthographic and phonological units [12]. Likewise, a neighboring site within the LP (supramarginal gyrus) mediates G-P conversion, i.e. the retrieval of the phonological codes for the letters to be read [15], [37], [51], while acting as a bridge to phonological processing in the IFG [29]. To be noted, we previously detected that the left supramarginal gyrus also yielded letter-selective activity during a passive viewing task [12]. DRC speaking, literature and present findings suggest that first, regular words would feed to the dorsal path (LP) for letter-selective mapping into phonological representation and in parallel, to the (posterior) ventral path for letter/sub-word analysis, reflecting cooperative/competitive dorsal/ventral interactions [2]. This view is in agreement with the DRC model arguing that regular words can be read in two ways. Despite parallel dorsal/ventral processing, only trafficking to the dorsal path (word pathway) is selective for words; this is further supported by the association between connectivity for this path (and not ventral path) and high reading performance (Figure 2, blue full arrow). Secondly, pseudowords would feed to the dorsal path for letter-selective mapping into phonological representation but not before the intervention of a substantial letter/sub-word analysis in the posterior ventral pathway (see [19], [20] for the important role of the region in bi- tri- or quadri-gram processing). The prominence of this posterior ventral pathway during pseudoword reading receives further support by the correlation of its connectivity with high reading performance (Figure 2, red full arrows).

### Limitations and alternative interpretations

Two points are to note about the words stimuli used here. First, we used regular words of high frequency and imageability thereby strengthening the reliance on the lexical vs. the non-lexical route [25]. However, irregular words are likely to rely on the lexical route to a higher extent; this should be interesting for further investigation. Second, it is important to note the relatively high degree of transparency in French. It would therefore make sense that words of a more opaque language (English) would rely more on the lexical (anterior LOT) pathway, and less on the dorsal G-P conversion (LP) as reported in the literature. It should be informative to address these points in the future using a similar design, but (i) between languages, and (ii) using irregular words.

We also note that our results could be interpreted by connectionist models (e.g. [52]) which represent orthography, phonology, and semantics as separate systems, presumably interacting via recurrent neural networks. This seems particularly pertinent given that our results point to a mechanism of parallel processing during reading. Nevertheless, there are several issues which led us to test our results rather according to the DRC model. First, the number of connections between the areas was mathematically limited and was thereby restricted to feed-forward connections given the posterior-to-anterior recruitment of the tested areas [12]. Second, our experimental design did not allow us to directly pinpoint a region for semantic processing, which should be addressed to account for connectionist models. Instead, our investigation focused on early and more basic processing stages of reading. The analysis of letter-strings (non-words) replicated the connectivity effect of pseudowords (vs. words) for the posterior reading triangle MOG→LOT→LP, thereby supporting the idea that it is implicated in visual-orthographic analysis whereas more anterior connections would involve subsequent processing steps such as phonological and semantic analyses. Altogether, the simplistic and parsimonious nature of the neuronal modeling applied here, together with the straightforward pattern of results, led us to consider the DRC model particularly suitable for accommodating our results.

Previous effective connectivity (e.g. [16]) and magneto-encephalography (e.g. [53]) accounts have reported distinct neuronal connections and dynamics during word and pseudoword reading. Given that these studies and ours used different tasks (e.g. oddball lexical decision vs. passive viewing) or applied the results on different anatomical models, they report different regions of interest, implying that word and pseudoword reading may in fact recruit a vaster neural network than the one reported in our study. In particular, because our connectivity model lacked a lexico-semantics ROI and focused on a restricted number of prominent regions during reading, it remains unclear which other regions participate in such complex process, and what recurrent loops and feed-back and -forward connections take place during reading. Nevertheless, the simplicity of our model is also its strength given that it straightforwardly reveals strong interactions between prominent (and serial) reading areas. In a similar vein, Seghier and colleagues [24] showed that posterior- and anterior-LOT networks may correspond to lexical and non-lexical reading, respectively. The authors explained their counter-intuitive finding by suggesting that higher activation may reflect more effort, not more efficiency. Although such possibility cannot be excluded, with respect to other network suggestions in the literature, we would rather suggest that numerous neural networks mediate reading, depending on stimulus fingerprint, language properties/characteristics, task demands and probably other factors. At present, it may not be possible yet to provide a clear and complete picture encompassing the totality of the neural networks underlying reading. However, we do contend that investigating neural networks should be more informative than investigating a brain region in isolation [54]. Studying interactions within neural networks thus contributes to elaborate the ever growing scope of understanding the processes underlying reading, especially when correlated to off-line performances. The core of our investigation was, therefore, not only to highlight pathways for which activity is selectively modulated by words and pseudowords, but also to expand upon previous reports by locating those that are liable to predict reading performance.

### Reading styles

Addressing the second point of our study, we found that (i) incongruent pathway reliance (word pathway vs. pseudoword pathway) as a function of the stimulus to be read predicts poorer reading performance (WRT/PWRT tests), (ii) congruent pathway reliance predicts higher reading skill (“*Alouette-R*” reading text reflecting skill in alternating pathway reliance), (iii) text reading was well predicted (85% to 93% of the variance) by an optimal combination of the preferential recruitment of the two pathways (especially the ability to use the pseudoword pathway). Overall, the results establish a firm link between reading performance and pathway reliance, in particular alluding to the prominent contribution of LOT, thereby lending support to (i) the recently observed correlations between LOT activation and reading performance in skilled readers [55], to (ii) the stronger modulatory effect on the LOT→LP feed-forward connection observed in skilled vs. impaired (children) readers during an orthographic/phonological conflicting rhyming task [29], and even to (iii) the correlation between gray matter volumes in this region and reading skills [56].

As a broader interpretation of the present results, we propose that the observed shifts in effective connectivity between word and pseudoword pathways are mediated through automatic mechanisms, which may rely on the extraction of the stimulus' global visual fingerprint and on a subsequent comparison with entries in the visual lexicon, so as to favor one of the routes. This idea was inspired by the early observation of reliance patterns (lexical vs. non-lexical) even in a population of skilled readers [57]. The particular nature of the text reading task used here, requiring high skill in rapidly alternating between lexical and non-lexical reading [31], led us to formulate the following interpretation: the efficiency with which readers alternate between one path to another reflects individualized ‘in cerebro reading styles’ (pathway reliance) and could, in turn, determine reading speed and proficiency. Such automated shifts in (lexical and non-lexical) reading styles [25] may be mediated through shifts in neural pathways [26], [27] on an unconscious level, as has been recently shown [28]. With respect to this view, we analyzed the data to define the efficient ‘in cerebro reading style’, i.e. the reliance pattern, predicting high performance during text reading. A (non-linear) trade-off between the word vs. pseudoword pathways (dark red on Figure 3), i.e. relying on the word pathway during word reading and on the pseudoword pathway during pseudoword reading, accounted best for high reading performance.

However, not all subjects followed the ‘efficient trade-off’. As an example of peculiar reading styles, subjects 1 and 2 were outliers in the multiple regression analysis (linear adjustment), and had highly negative Delta values (Table 4), i.e. they exhibited higher connectivity values for the non-lexical than the lexical route not only during pseudoword reading but also during word reading. Such an ‘abnormal’ connectivity pattern could be taken as an indication of an overuse of the non-lexical route regardless of stimulus-compliance to G-P conversion rules. Such a strategy has been recently observed in deaf readers [58] but seems to extend here, even to clinically normal readers. Interestingly, the other subject whose data did not conform to the model (discarded subject 15) also revealed a peculiar reading style, but this time reversed, i.e. this subject exhibited highly positive Delta values (Figure 3, right panel; Table 4). In this case, this indicates an overuse of the lexical route. Interestingly, although subject 15 did not fit the connective model during pseudoword reading (p = 0.04, Table S2), this subject's performance perfectly fit the connective model during word reading (p = 1, Table S3). This also indicates that our model relies more heavily on the non-lexical route: although outliers in the regression, subjects 1 and 2 did fit the overall model whereas subject 15 did not. Looking at the scores, subjects 15 and 1 were also the most skilled readers (highest CTL value; Table 4) whereas subject 2 had very poor reading skills (lowest CTL value). This suggests that the high performance is obtained with a (non-linear) trade-off between the lexical vs. non-lexical routes (dark red on Figure 3, subjects 4, 5 and 6, Table 4) but also that the ‘quasi-exclusive’ use of one route or the other can elicit highly-efficient (subjects 1 and 15) or inefficient (subject 2) performances. These peculiar reading styles did not conform to our model/regression and are illustrated in Figure 3 (right panel). In a nutshell, our findings are in agreement with data suggesting individual reading styles/profiles that can be accounted for by the DRC model [59]. Furthermore, the observation of the ‘quasi-exclusive’ use of paths may suggest that subjects 1 and 15 may have relied on alternative pathways that are not included in our model, which would explain their excellent reading performance. By contrast, subject 2 may have not resorted to alternative pathways, thereby resulting in poor reading performance. Future research applying more complex connectivity models on reading data may reveal that different individuals rely on different pathways within a vast and complex neural network during reading.

Likewise, these observations raise the possibility that individualized reading styles may be explained by factors other than connectivity measures such as education, age and gender. To this aim, we found that the variance in reading skill could also be explained by the number of years of education (linear correlation: r = .55 with WRT, r = .54 with PWRT and r = .61 with CTL indices; p<.05) but not demographic data (age and gender). More importantly, the number of years of education did not correlate with delta word (r = 0.02) or delta pseudoword (r = −0.29), suggesting an independent contribution of the number of years of education (practice) and ‘in cerebro’ reading styles. This finding is of particular interest given that lexical/non-lexical reading styles are already observed in childhood [60] and may be established during school time by individualized reading instruction methods [61]. Hence, the present work may be used in future to elaborate the scope of understanding both early reading acquisition and the influence of various factors on reading skill.

Most recently, Seghier and colleagues [24] showed that among a population of skilled readers, some rely more on lexical than sub-lexical reading and vice-versa (out-of-scanner assessment of irregular word and pseudoword reading), and that this correlated with the activation of two neural networks (fMRI for word reading). In the present study however, we directly focus on the lexical and sub-lexical pathways as assessed by connectivity during word and pseudoword reading in the scanner, and correlate it to reading tests outside the scanner. Although the studies applied different tasks and approach, and therefore investigated different neural networks, we contend that both are complementary in that they strengthen the idea that skilled readers differ by relying on different reading styles which in turn reflect activation of different neural networks.

To conclude, the results here reveal, for the first time, functional paths in the brain that are selectively involved in word and pseudoword reading in a manner consistent with the DRC model. Overall, the findings (i) confirm prior neuropsychological data by providing a remarkably good neural account of the early processing (visuo-orthographic) of the two routes in the DRC model, (ii) extend the knowledge about the non-lexical route, showing a mediation via the posterior LOT, (iii) demonstrate that incongruent or congruent reliance on these pathways predicts poor or skilled reading, (iv) raise the novel idea of efficient ‘in cerebro’ reading style depending on the stimulus to be read (known word vs. unknown word) and argue that individually unique reading styles may translate to either skilled or deficient reading ability, (v) and finally, introduce a link between individual reading styles and reading proficiency, which could be in part established during school time by individualized reading instruction methods [61]. The findings here could also be considered as a first demonstration of the pattern of cerebral information trafficking which one ‘should follow’ in order to yield high reading performance. This may be of particular interest for applications already during school time for reading difficulties, such as in the case of developmental dyslexia.

## Methods

In the present manuscript we extended the analyses performed on data from our previous experiment [12].

### Participants

Fifteen healthy individuals (eight females, mean age 27.3, sd 3.4 years), all university students (5–8 years) with normal or corrected-to-normal vision participated in this experiment. All were right-handed on the Edinburgh handedness inventory, native French speakers, and free from any history of neurological or psychiatric illness or medical treatment.

### Ethics Statement

The Toulouse INSERM (Institut national de la santé et de la recherche médicale) ethics committee approved the experimental protocol and informed written consent was obtained from the subjects after the nature and possible consequences of the study had been explained to them.

### Out-of-scanner reading assessment

Subjects' reading ability was investigated outside the scanner by applying the Word reading Response Time (WRT) and the Pseudoword reading Response Time (PWRT) tests, that have been repeatedly used in our group for the diagnosis of developmental dyslexia in adults (e.g. [30], [62], [63]). In these tests, four blocks of words and pseudowords (twenty each) were presented in an ABBA design on a computer screen; naming latency was recorded via a voice-key and reading correctness was registered. The words used were highly concrete and familiar nouns. Pseudowords maintained words' ‘envelope’ but with different internal consonants. Participants were asked to read each word/pseudoword as soon as it appeared on the screen. Once the subject had responded and the latency had been recorded via a voice key, the word disappeared; there was a 1-second interval before the next stimulus was presented. Voice onset time for single word/pseudoword reading was transformed into a z-score (z = -1*(vo – avg)/sd; ‘vo’ is voice onset, ‘avg’ and ‘sd’ are the average and the standard deviation of the population reference) while simple reaction times for a dot stimulus provided a baseline. Hence, positive z-values reflect higher performance whereas negative z-values reflect lower performance in comparison to the general population. Note that, spelling accuracy and voice onset in these two tests, both reflect reading impairment [62]. Furthermore, they convey an indirect estimation of the efficiency of the lexical and non-lexical routes, i.e. low scores in the first test (WRT) would point to a deficient utilization of the lexical route [32], whereas low scores in the second (PWRT) may reflect a damaged reliance on the non-lexical route [33]. Thus, we expected negative correlation between scores in these tests and incongruent reliance of pathways, i.e. reliance on the ‘word pathway’ (posterior dorsal stream) during pseudoword reading and ‘pseudoword pathway’ (posterior ventral stream) during word reading. To expand our investigation to the reading circumstances closer to every-day life, we also used the “*Alouette-R*” test [31], a standardized test for reading text in French which at variance with the former two tests, directly assesses text reading speed and precision (CTL index). This time-limited text involves both frequent words, and very rare words (making them appear as pseudowords to almost any subject), as well as words having low probability provided the sentence context in which they take place. The particular contents of this text requires the subject to alternate quickly between ‘standard’ text reading involving mainly the lexical route and the non-lexical route in order not to misprocess the ‘catch-up’ words hidden in the text from place to place. Note that whereas the first two tests are markers of impairment in word or pseudoword reading, i.e. predict negative correlation with pathway reliance, “*Alouette-R*” test assesses proficiency at dealing fluently with both routes during text reading. We therefore hypothesized a positive correlation between CTL scores and congruent reliance of pathways, i.e. reliance on the word pathway during word reading and vice versa.

### fMRI stimuli

Stimuli were all embedded in pseudo-characters so as to maintain a constant string length (seven characters), and displayed on a grey (RGB: 160, 160, 160) background to avoid visual fatigue (see example of stimuli in Figure S1). Two hundred and eight stimuli were designed, twenty six per stimulus category, thus matching their frequency of appearance. Stimuli were matched (intra-category) and distinguished (inter-category) for their angularity, visual surface and complexity, orthographic units in general and consonant and vowel structure in particular, phonological and lexical-semantic units, mean frequency of appearance for words, mean positional letter frequency (MPLF), mean positional bigram frequency (MPBF) and mental imagery score for words.

### Tasks and procedures

Subjects were briefly trained and familiarized with the procedures and stimuli prior to fMRI scanning. During the scanning, stimuli were displayed via a dual-display stereoscopic video projector (VisuaStimDigital, Resonance Technology Inc.) in synchrony with functional acquisition that duplicated the experimental computer screen with 500,000 pixels per 0.25 square inch resolution and a refresh rate of 85 Hz. In order to minimize ocular saccades and numerous fixations at different string positions, stimuli were presented with a horizontal visual angle of 4.2° for 200 ms. Additionally, the maximal number of letters was limited to five thus facilitating stimulus recognition in one fixation, although through the whole experiment the total length was always of 7 characters if one counts the pseudo-character flankers.

Participants were passively exposed to blocks of stimuli during five runs of five minutes each. Each run contained ten 17-s long blocks of stimuli that alternated with 12.5-s long blocks of visual fixation (fixation-cross of 0.65° visual angle). Blocks were presented pseudo-randomly to increase condition (stimulus category) alternation and avoid condition repetition among successive blocks. Each of the eight conditions was repeated in six different blocks among the five runs in such an order as to avoid interference with the low frequencies of scanner noise and physiological rhythms. In the fifth run, the last two blocks were used to equalize run-length, but discarded from analysis, so as to maintain an equal number (six) of blocks per condition. Each block contained twelve different stimuli of the same condition with a random inter-stimulus interval (ISI) ranging from 600 to 1100 ms so as to avoid stimulus anticipation or rhythmic activity and to maximize the BOLD signal [64]. This also allowed us to sample data in a distributed way over the ISI, eschewing a possible bias of estimated activation [65]. At the end of each run, subjects could rest for 2–3 minutes and were asked to report stimulus visibility or any other difficulties or problems that could bias the experiment.

### fMRI parameters

All subjects were scanned at the Neuroradiology service of Toulouse Purpan Hospital on a 1.5 Tesla Siemens Magnetom Vision scanner (Erlangen, Germany) equipped for multi-slice echo-planar imaging (EPI). For functional MRI runs, blood oxygen level-dependent (BOLD) imaging was performed using a T2*-weighted single-shot EPI sequence (60 ms echo time (TE), 2430 ms repetition time (TR), 90° flip angle, 250 mm field of view (FOV), 64×64 acquisition matrix with 16 interleaved slices parallel to the intercommissural plane (from z = −35 to z = 45), 3.91×3.91×5 mm voxel size). The high-resolution anatomical scan was acquired on the same plane as the EPI data at the end of the functional runs using a 3D MPRAGE sequence (TE = 4 ms, flip angle = 8°, FOV = 300 ms 5/8, 160×256 matrix, 1.17×1.17×1.18 mm voxel size).

### Image processing

All functional images were pre-processed using techniques implemented in Statistical Parametric Mapping (SPM2, Welcome Department of Cognitive Neurology, http://www.fil.ion.ucl.ac.uk). The functional scanning sessions contained 123 acquisition volumes, of which the first four were discarded for signal stabilization. A slice timing correction was performed with the fifteenth slice (the middle temporal one) as the reference. The sixtieth volume of the prior 3D-session was used as a reference for realignment of functional images to correct for head motion. T1-weighted anatomical images were coregistered to the mean EPI image, and were used for the normalization of functional images onto the Montreal Neurological Institute T1-template with a resampling at 2 mm^3^ (5th degree B-Spline interpolation).

### fMRI data analysis

Images were smoothed with a 6-mm-at-full-width-half-maximum Gaussian filter ensuring data normality. For each subject, the spatially normalized and smoothed images were used to create eight condition-type images per subject (general linear model with one regressor per condition and session convolved with a box car function) and entered into an ANOVA model to inquire commonalities and differences between conditions [12]. Figures illustrate statistical parametric maps overlaid on the individual ‘inflated’ Colin brain atlas anatomical images [66].

Functional MRI time points (TR = 2430 ms) were extracted from each individual data set with spheres (3×3×3 mm) centered at cluster maxima-coordinates of the four left-hemisphere ROIs revealed in our analyses: (i) the left middle occipital gyrus (-32 -91 10), (ii) the left lateral occipital-temporal area (-46 -68 -5), (iii) the left parietal (-24 -50 43), and (iv) the left inferior frontal gyrus (-51 18 14). Each condition was repeated in six functional blocks, each one consisting of twelve time points (seven for condition, five for fixation), thus resulting in 72 concatenated time points for most but not all conditions. Hence, to balance vector length among conditions, we selected 64 time points per condition for each individual in each region. Note that, each condition block was multiplied by the first eleven (out of twelve) time points of the hemodynamic response function so as to maximize signal extraction and to avoid interference with the subsequent block. Finally, data were high-pass filtered (cut-off frequency 0.05 Hz) to remove low-frequency concatenation-generated signals.

Effective connectivity was then assessed by means of structural equation modeling (SEM) implemented in LISREL software (version 8) [67]. After averaging the observed data (BOLD signal) across subjects, covariance matrices were computed for both the observed data and for the estimates of the theoretical model. A robust estimator of maximum likelihood is achieved by an iterative procedure of adjusting the predicted values with the observed values, resulting in a β-coefficient value for each connection. Residual variance representing unmeasured influences from outside the model is also incorporated for each connection. It reinforces the statistical power and the precision of the calculated β-coefficient values. The null hypothesis postulates no difference between the predicted and the observed matrix. Thus, path models which provided a good account of the observed data were indicated by the impossibility of rejecting the null hypothesis (p>0.05); likewise a good fit of the model corresponded to low values (p<0.1) for the root mean square error of approximation (RMSEA). β-values reflect path strength; more precisely, for a given connection A→B, a positive β-value would mean that region A exerts a positive modulatory effect on region B, i.e. it increases the activity of region B. Alternatively, a negative β-value would mean that region A exerts a negative modulatory effect on region B, i.e. it decreases the activity of region B.

To define a path model that would account for the pattern of cortico-cortical associations during the passive viewing of all the eight conditions, we used our prior knowledge about prominent reading areas and the posterior-to-anterior fashion in which they are recruited [12]. Following the principle of parsimony, we started by first testing an economical model (few connections) and then making it more complex by adding new ROIs, forward and backward connections. The objective was to retain the fewest ROIs (variables) and paths that explain as much as the variance in the phenomenon is possible [68]. Specifying paths and β-coefficient values (path strength), we retained the following explanatory variables (ROIs) and the most relevant paths between them which best accounted for the observed fMRI data: Left MOG to LOT, Left MOG to LP, LOT to LP, LOT to IFG and LP to IFG. Among different tested models which could represent simpler or more complex dynamics, this parsimonious and unidirectional model provided the best fit for observed data during the passive viewing of eight different pseudo-linguistic and linguistic stimulus categories (see high P-values in Table S1). Additionally to this, the chosen model consisted of four ROIs therefore allowing for the estimation of a maximum number of six (4×3/2) variables. Thus, we have here estimated five variables (paths). Finally, beside the fact that adding more connections than those already present would statistically render the model unstable, it is also known that adding double-sense connections in SEM is to be avoided at the risk of destabilizing the model's fit.

Hence, our choice of important connections had to be limited and was therefore inspired by the posterior-to-anterior fashion in which prominent reading areas are recruited (c.f. [12] for a direct or [8], [34], [35], [36] for an indirect demonstration), thus retaining unidirectional forward paths in the model. It is important to note, however, that the potential contribution of feed-back connections and that of other reading areas is overlooked in the present account. Mitigating this concern, the temporal resolution of an fMRI measure, which is of about a couple of seconds, has for consequence that the weight of a directional path is in fact the net result of the time integral of all the millisecond-range information circulating (forwards and backwards) from within the duration of the measurement. In that sense, the weight of the oriented path depends on bi-directional information. Recapitulating upon bidirectional concerns, we argue that the more constrained the model, the more robust, powerful and interpretable the results are. Thus, investigating reading using a parsimonious, yet functionally robust, forward model should highlight important information trafficking during reading.

To test for differences between words and pseudowords on the paths within the network, a stacked model approach was used. This approach consists in comparing a ‘free’ model in which all paths are allowed to vary between two conditions, to a ‘restricted’ model in which the tested path is forced to be equal across conditions. In a first analysis, we compared pathways for each reading condition thus revealing which pathways are more ‘engaged’ than others. In a second analysis, we compared reading conditions for each pathway, thus revealing which pathway is preferentially used during the reading of various stimuli.

To test for reading proficiency, Spearman rank correlation coefficients (p<0.05 - Table 3) were computed between on the one hand, z-scored Word Reading Test (WRT) and Pseudoword Reading Test (PWRT) accuracy scores [18], and “*Alouette-R*” CTL indices [31], and on the other hand, BOLD path coefficients during word and pseudoword reading (Tables 2,S2,S3 – Figure 2). P-values were adjusted for multiple comparisons by controlling the false discovery rate (FDR) [69]. Voice onset and spelling errors in the first two tests (WRT and PWRT) constitute a valid criteria for the diagnosis of reading impairment [32], [33], [62], [63]. Low performance in these tests for poor readers (dyslexics) but not for good readers (healthy subjects) (e.g. ref [62]) may be due to an erroneous reliance on the reading neural network, in particular in the parieto-temporal region [70]. These observations thus motivated the rationale hypothesizing that scores in WRT and PWRT tests should anti-correlate with incongruent reading pathway, which could in turn imply a tendency for reduced reading performance. Noteworthy, these observations could not rule out the possibility that these tests could also positively correlate with congruent reading pathway. Nevertheless, for the purpose of predicting reading skill (and not reduced reading performance) we reasoned the “*Alouette-R*” test more adequate. “*Alouette-R*” test is particularly conceived to assess proficiency at rapidly alternating between lexical and non-lexcial processing during text reading [31]. We formulated two working hypotheses: (i) scores in WRT PWRT should negatively correlate with incongruent reliance of pathways, i.e. reliance on the word pathway during pseudoword reading and vice versa, and (ii) CTL (“*Alouette-R*”) scores should positively correlate with congruent reliance of pathways, i.e. reliance on the word pathway during word reading and on the pseudoword pathway during pseudoword reading.

In addition to the above analysis, the difference (Delta) between connectivity values of MOG→LP and MOG→LOT were computed and correlated with reading skill (CTL indices) using Spearman rank correlation coefficients and multiple regression analyses. The latter analyses aimed at testing whether the ‘preferential use’ of the congruent route could predict individual's reading skill. A 1^st^ analysis revealed that there were 2 outliers. These subjects were identified using absolute z-residues. Z-residues correspond to the absolute value of the standardized residuals, i.e. the ratio between the centered value of residuals (that is, the difference between the measured value and the estimated value) and its standard deviation; it is thus equivalent to a z-value. Subjects with z-residues (absolute values) greater than 2 reflect a deviation of approximately two standard deviations from the mean value of the population and are therefore considered as outliers (subjects 1 and 2) (see Figure S2). A second analysis was then performed using only 12 subjects. Finally, an augmented model was tested, using not only delta word and delta pseudoword but also age, gender and the number of years of education. The two models were compared using an F test to examine if those variables explained the data better.

## Supporting Information

Figure S1 Examples of stimuli used for each of the eight experimental stimulus-categories. Stimuli were all embedded in pseudo-characters so as to maintain a constant string length.
(2.05 MB TIF)

Figure S2 CTL z-residues as a function of subject number. Identified outliers are marked with red circles.
(0.19 MB TIF)

Table S1 Group β-values of effective connectivity according to stimulus category.
(0.06 MB DOC)

Table S2 Individual β-, p- and RMSEA values of effective connectivity during pseudoword reading.
(0.07 MB DOC)

Table S3 Individual β-, p- and RMSEA values of effective connectivity during word reading.
(0.07 MB DOC)
