# Supplementary figures and images for: Testing for the Dual-Route Cascade Reading Model in the Brain: An fMRI Effective Connectivity Account of an Efficient Reading Style
Source: PLoS One. 2009 Aug 18;4(8):e6675. doi: 10.1371/journal.pone.0006675 (PMC2724737; doi:10.1371/journal.pone.0006675)

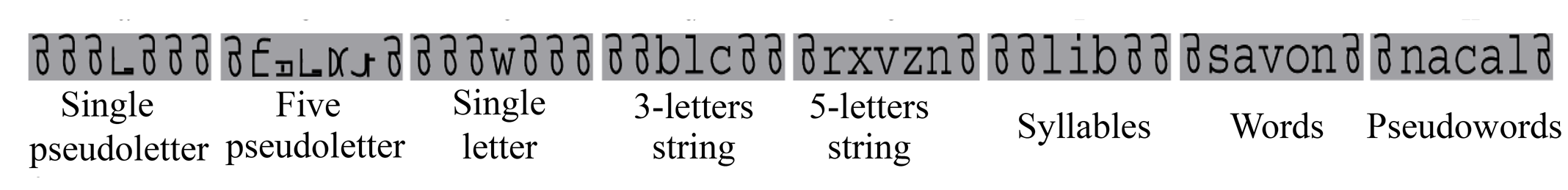

Supplement: Figure S1 — Examples of stimuli used for each of the eight experimental stimulus-categories. Stimuli were all embedded in pseudo-characters so as to maintain a constant string length. (2.05 MB TIF) [file pone.0006675.s001.tif]

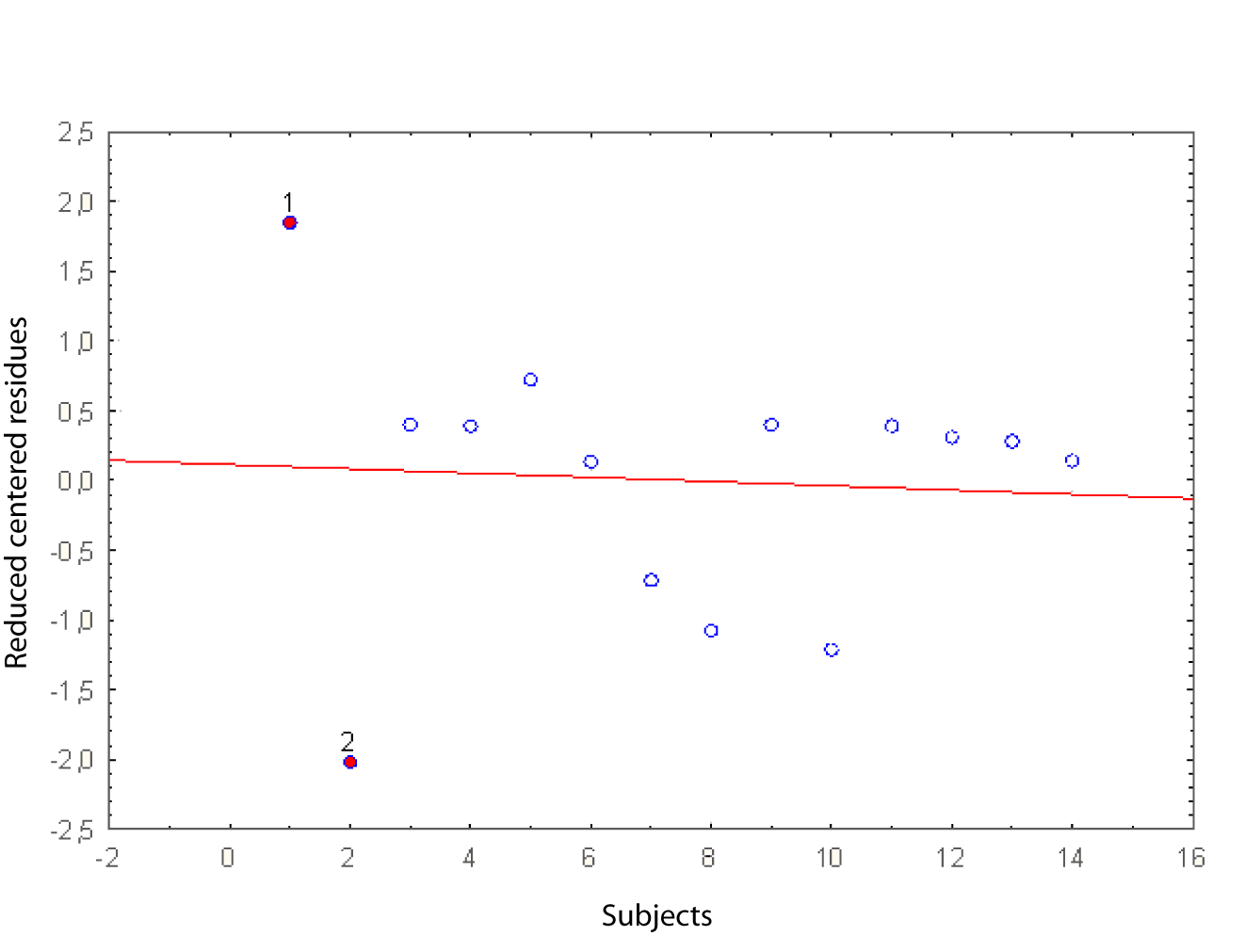

Supplement: Figure S2 — CTL z-residues as a function of subject number. Identified outliers are marked with red circles. (0.19 MB TIF) [file pone.0006675.s002.tif]
