# Supplementary material for: Testing for the Dual-Route Cascade Reading Model in the Brain: An fMRI Effective Connectivity Account of an Efficient Reading Style
Source: PLoS One. 2009 Aug 18;4(8):e6675. doi: 10.1371/journal.pone.0006675 (PMC2724737; doi:10.1371/journal.pone.0006675)
Supplement: Table S1 — Group β-values of effective connectivity according to stimulus category. (0.06 MB DOC) [file pone.0006675.s003.doc]

**Supporting Information**

**Table S1.** Group β-values of effective connectivity according to stimulus category.

|  | ***MOG → LOT*** | ***MOG → LP*** | ***LOT → LP*** | ***LOT → IFG*** | ***LP → IFG*** | ***p- value*** | ***RMSEA*** |
| --- | --- | --- | --- | --- | --- | --- | --- |
| *Single-pseudoletters* | 0.62 | 0.48 | 0.16 | 0.03 | 0.12 | 0.617 | 0 |
| *5-pseudoletters* | 0.71 | 0.23 | 0.24 | 0.29 | 0 | 0.999 | 0 |
| *Single-letters* | 0.6 | 0.17 | 0.2 | 0.21 | 0.09 | 0.694 | 0 |
| *3-letter Strings* | 0.55 | 0.31 | 0.15 | 0.2 | 0.23 | 0.97 | 0 |
| ***Non-words*** | 0.83 | -0.04 | 0.71 | 0.02 | 0.36 | 0.72 | 0 |
| *Syllables* | 0.68 | 0.63 | -0.03 | -0.03 | 0.18 | 0.895 | 0 |
| ***Words*** | 0.44 | 0.43 | -0.2 | 0.11 | 0.24 | 0.996 | 0 |
| ***Pseudowords*** | 0.75 | -0.04 | 0.55 | 0.29 | 0.55 | 0.685 | 0 |

Path models which provided a good account of the observed data were associated with small minima of the discrepancy function F and correspondingly large probabilities (p > 0.05) under the null hypothesis, as well as with low values (p < 0.1) for the root mean square error of approximation (RMSEA). Categories used for the present study are presented in **bold**. **MOG,** left middle occipital gyrus; **LOT**, left occipito-temporal junction; **LP**, left parietal cortex; **IFG**, left inferior frontal gyrus.
