# Supplementary material for: Testing for the Dual-Route Cascade Reading Model in the Brain: An fMRI Effective Connectivity Account of an Efficient Reading Style
Source: PLoS One. 2009 Aug 18;4(8):e6675. doi: 10.1371/journal.pone.0006675 (PMC2724737; doi:10.1371/journal.pone.0006675)
Supplement: Table S2 — Individual β-, p- and RMSEA values of effective connectivity during pseudoword reading. (0.07 MB DOC) [file pone.0006675.s004.doc]

**Table S2.** Individual β-, p- and RMSEA values of effective connectivity during **pseudoword reading**.

|  | **MOG → LOT** | **MOG → LP** | **LOT → LP** | **LOT → IFG** | **LP → IFG** | **p-value** | **RMSEA** |
| --- | --- | --- | --- | --- | --- | --- | --- |
| 1 | 0.40 | 0.24 | 0.33 | 0.19 | 0.37 | 1 | 0 |
| 2 | 0.44 | 0.09 | 0.35 | 0.27 | 0.12 | 0.83 | 0 |
| 3 | 0.50 | 0.16 | 0.12 | 0.14 | 0.40 | 0.98 | 0 |
| 4 | 0.47 | 0.14 | -0.01 | 0.58 | 0.26 | 1 | 0 |
| 5 | 0.73 | 0.22 | 0.16 | 0.47 | 0.18 | 0.86 | 0 |
| 6 | 0.48 | 0.01 | 0.47 | 0.02 | 0.43 | 0.22 | 0.08 |
| 7 | -0.05 | 0.13 | 0 | -0.07 | -0.17 | 0.93 | 0 |
| 8 | 0.32 | 0.50 | 0.07 | 0.09 | 0.53 | 0.29 | 0 |
| 9 | 0.22 | -0.05 | 0.24 | 0.2 | -0.11 | 0.79 | 0 |
| 10 | 0.24 | 0.58 | -0.43 | -0.21 | 0.12 | 1 | 0 |
| 11 | 0.52 | 0.11 | 0.15 | 0.38 | 0.19 | 0.10 | 0.12 |
| 12 | 0.34 | -0.11 | -0.04 | 0.1 | 0.31 | 0.68 | 0 |
| 13 | 0.64 | 0.48 | 0.15 | 0.04 | 0.61 | 0.96 | 0 |
| 14 | 0.02 | 0.11 | 0.27 | 0.14 | 0.37 | 0.84 | 0 |
| 15 | -0.13 | 0.01 | 0.09 | 0.26 | -0.02 | 0.04 | 0.14 |

**MOG**, left middle occipital gyrus; **LOT**, left occipito-temporal junction; **LP**, left parietal cortex; **IFG**, left inferior frontal gyrus; **RMSEA**, root mean square error of approximation.
