# Supplementary material for: Testing for the Dual-Route Cascade Reading Model in the Brain: An fMRI Effective Connectivity Account of an Efficient Reading Style
Source: PLoS One. 2009 Aug 18;4(8):e6675. doi: 10.1371/journal.pone.0006675 (PMC2724737; doi:10.1371/journal.pone.0006675)
Supplement: Table S3 — Individual β-, p- and RMSEA values of effective connectivity during word reading. (0.07 MB DOC) [file pone.0006675.s005.doc]

**Table S3.** Individual β-, p- and RMSEA values of effective connectivity during **word reading**.

|  | **MOG → LOT** | **MOG → LP** | **LOT → LP** | **LOT → IFG** | **LP → IFG** | **P-value** | **RMSEA** |
| --- | --- | --- | --- | --- | --- | --- | --- |
| 1 | 0.51 | 0.24 | 0.30 | -0.01 | 0.52 | 0.26 | 0.07 |
| 2 | 0.44 | 0.21 | 0.37 | -0.19 | 0.28 | 0.06 | 0.13 |
| 3 | 0.37 | 0.12 | 0.25 | 0.23 | -0.02 | 0.83 | 0 |
| 4 | 0.25 | 0.37 | -0.17 | 0.54 | 0.14 | 1 | 0 |
| 5 | 0.24 | 0.42 | 0.26 | 0.05 | 0.25 | 0.63 | 0 |
| 6 | 0.15 | 0.11 | 0.34 | 0.06 | 0.12 | 1 | 0 |
| 7 | 0.33 | 0.13 | -0.01 | 0.14 | -0.2 | 0.80 | 0 |
| 8 | 0.28 | 0.30 | 0.34 | 0.56 | 0.08 | 0.99 | 0 |
| 9 | 0.46 | 0.16 | 0.12 | 0.21 | -0.05 | 1 | 0 |
| 10 | 0.46 | 0.03 | 0.43 | 0.17 | -0.06 | 0.92 | 0 |
| 11 | 0.37 | 0.29 | -0.02 | 0.09 | 0.24 | 0.60 | 0 |
| 12 | 0.36 | 0.29 | -0.03 | 0.25 | 0.07 | 0.05 | 0.14 |
| 13 | 0.38 | 0.31 | 0.48 | -0.04 | 0.37 | 1 | 0 |
| 14 | 0.38 | 0.08 | 0.14 | 0.27 | 0.20 | 1 | 0 |
| 15 | 0.26 | 0.40 | -0.12 | 0.05 | -0.05 | 1 | 0 |

**MOG**, left middle occipital gyrus; **LOT**, left occipito-temporal junction; **LP**, left parietal cortex; **IFG**, left inferior frontal gyrus; **RMSEA**, root mean square error of approximation.
